# Prevention of: self harm in British South Asian women: study protocol of an exploratory RCT of culturally adapted manual assisted Problem Solving Training (C- MAP)

**DOI:** 10.1186/1745-6215-12-159

**Published:** 2011-06-21

**Authors:** Nusrat Husain, Nasim Chaudhry, Steevart V Durairaj, Imran Chaudhry, Sarah Khan, Meher Husain, Diwaker Nagaraj, Farooq Naeem, Waquas Waheed

**Affiliations:** 1 The Lantern Centre, Lancashire Care NHS Foundation Trust, Vicarage Lane, Fulwood, Preston, Lancashire PR2 8DY, UK

## Abstract

**Background:**

Suicide is a major public health problem worldwide. In the UK suicide is the second most common cause of death in people aged 15-24 years. Self harm is one of the commonest reasons for medical admission in the UK. In the year following a suicide attempt the risk of a repeat attempt or death by suicide may be up to 100 times greater than in people who have never attempted suicide.

Research evidence shows increased risk of suicide and attempted suicide among British South Asian women. There are concerns about the current service provision and its appropriateness for this community due to the low numbers that get involved with the services. Both problem solving and interpersonal forms of psychotherapy are beneficial in the treatment of patients who self harm and could potentially be helpful in this ethnic group.

The paper describes the trial protocol of adapting and evaluating a culturally appropriate psychological treatment for the adult British South Asian women who self harm.

**Methods:**

We plan to test a culturally adapted Problem Solving Therapy (C- MAP) in British South Asian women who self harm. Eight sessions of problem solving each lasting approximately 50 minutes will be delivered over 3 months. The intervention will be assessed using a prospective rater blind randomized controlled design comparing with treatment as usual (TAU). Outcome assessments will be carried out at 3 and 6 months. A sub group of the participants will be invited for qualitative interviews.

**Discussion:**

This study will test the feasibility and acceptability of the C- MAP in British South Asian women. We will be informed on whether a culturally adapted brief psychological intervention compared with treatment as usual for self-harm results in decreased hopelessness and suicidal ideation. This will also enable us to collect necessary information on recruitment, effect size, the optimal delivery method and acceptability of the intervention in preparation for a definitive RCT using repetition of self harm and cost effectiveness as primary outcome measures.

**Trial Registration:**

Current Controlled Trials 08/H1013/6

## Background

Prevention of suicide is a priority for health services in England [1]. Among the ethnic minority groups in UK there are reports of high rates of suicide in South Asian women [2]. Studies done previously in the UK similarly suggest that rates of self harm in British South Asian women are higher compared to the white population [3,4]. Of the known risk factors for suicide, the most strongly predictive of a future suicide attempt or completed suicide, are hopelessness and a prior history of self harm [5]. In Manchester data collected in Emergency departments over a four-year period showed a higher rate of self harm in South Asians than white European females (relative risk 1.5 times in 16-24 years age group). Interpersonal conflicts within the family were the main precipitating factor, which were significantly more frequent in South Asians than in white females. In spite of no differences in clinical presentation and suicidal intent, there were significant differences in the management of the two groups. Referral back to primary care was the most common management plans for South Asians, while European whites were referred to psychiatric services for further management [6,7].

In our previous study in primary care (Husain et al, 1997) and in the population based study [8] concerning the mental health needs of people of Pakistani family origin, we reported that women of Pakistani origin living in the north west of England have high rates of emotional distress and depression. A total of 13.8% of the 475 Pakistani women reported suicidal ideation on Self Rating Questionnaire (SRQ). This was associated with increased psychological distress (mean SRQ score of 12.3 in participants with suicidal ideas as compared to 5.23 in those who did not report suicidal ideas) [9]

Studies in the UK have reported socio- cultural determinants as contributing factors for depression such as cultural conflict within families particularly around family values, conflict in areas such as personal choices in relationships, jobs, pressure from family to conform to arranged marriages [10]. A previous qualitative study has demonstrated that young South Asian women access care only in times of crisis as a last resort and not early on when problems initially start [11]. It is suggested that services fail to engage with ethnic minorities [12]. Following an episode of self harm due to lack of appropriate treatment offered or a negative impact of services British South Asian women may feel reluctant to present again to services after a further self harm episode. The higher rate of suicide in young women of South Asian origin may be related to untreated self harm [13]

Self harm in British South Asian women may be due to their culturally related psychosocial stressors, there is evidence in literature that problem solving therapies [14] and interpersonal psychotherapy [15] has helped those who self harm. These therefore may also help women of British South Asian origin. The interventions will have to be adapted to meet the specific needs of this group. As most episodes of self harm are following a family conflict there is a strong case for demonstrating the effectiveness of an intervention which is able to address such cultural specific issues.

Problem solving therapy has a sound theoretical basis. Consistent evidence has shown that people who attempt suicide have poor problem solving skills [16]. It is hypothesized that problem-solving deficits contribute to hopelessness and depression, which in turn increase the probability of suicidal ideation and intent [17]. Problem solving therapy has been found to be effective in reducing levels of depression and hopelessness in patients who have attempted suicide [18]. In addition problem-solving therapy is a brief, cost effective, pragmatic intervention that has the potential to be widely utilized in clinical practice. Thus a strong rationale exists for the exploratory trial of problem solving therapy in order to clearly determine the acceptability of this intervention following self harm in British South Asian population.

### Aims and objectives

In preparation for a future definitive randomized control trial using repetition of self harm and cost effectiveness as the outcome measures, in this pilot trial we aim to collect necessary information on the assessment instruments, effect size, the optimal delivery and acceptability of the intervention. We also want to gauge the optimum length of treatment and to evaluate the therapy in a preliminary way to see if we can determine a treatment effect.

A further aim is to determine whether C- MAPS compared with treatment as usual results in decreased hopelessness and suicidal ideation. We will explore the impact of the intervention on patient's resolution of interpersonal and family problems to fully understand the mechanism of action of C- MAPS.

## Methods

We will conduct a pilot randomized controlled trial of culturally adapted manual assisted Problem Solving Therapy (C- MAPS) in British South Asian females who self harm. Ethical approval was obtained from Tameside &Glossop Local Research Ethics Committee for the study (REC Reference number: 08/H1013/6). The intervention will be assessed using a prospective rater blind randomized controlled design, with an experimental and a control treatment as usual (TAU) group. Face to face baseline interviews followed by 3 months outcome assessments will be carried out. Further six months follow up assessments will be through post or telephone. A sub group of participants will be invited for in depth qualitative interviews exploring about barriers accessing mental healthcare and family dynamics.

### Inclusion Criteria

All 16-65 years old self ascribed British South Asian women presenting to emergency department or at the general practices after an episode of self-harm will be invited to participate in the trial. Participants should be resident in the trial site catchment area and be able to communicate in Urdu, Hindi, Punjabi or English.

Self harm will be defined as self inflicted injury, and/or ingestion of drugs in excess of the recommended therapeutic dose, with some intention of ending one's life [19]

### Exclusion Criteria

Temporary resident unlikely to be available for follow up, those fulfilling the ICD 10, Research diagnostic criteria for Organic brain disorders, Alcohol and drug dependence, Schizophrenia and Bipolar Affective Disorders, and patients who require psychiatric admission after an episode of self-harm will be excluded from the study.

### Trial Centers & Proposed sample size

East Lancashire and Greater Manchester have a large South Asian population [20]. From the case records we know that around 100 patients of South Asian origin present to the emergency departments/GP practices over a 12 months period. We propose to recruit enough patients so that we have at least 10 patients in the experimental group.

### Recruitment plan

The study will be conducted at the emergency departments of Royal Blackburn, North Manchester General and Manchester Royal Infirmary hospitals and GP practices that cover areas with large South Asian population. We will be using culturally appropriate strategies to promote the trial. Liaison with the community has already been initiated through awareness talks in partnership with the voluntary sector organizations. These voluntary organizations were also invited to comment on the design of the trial and content of the intervention. Information about the study in the form of posters and information sheets in the four study languages (Urdu, Hindi, Punjabi and English) will be made available. The therapists in the team are also proficient in these south Asian languages. The research team will also conduct regular liaison meeting with the emergency department, crisis team staff and GP practices to promote the study within the departments.

Consecutive patients meeting inclusion criteria will be informed about the study by the clinician who will take verbal or written consent for the research team to make the contact.

The study will be explained to each participant in detail by a researcher and written consent will be obtained. Family participation will be encouraged but only with participants' permission. For randomization the researcher will refer to an allocation sequence that will be provided by the offsite trial statistician and based on a computer generated list of random numbers. Even if there is more than fifty percent refusal we expect sufficient number of patients each assigned to C- MAPS or the treatment as usual group.

### Description of intervention

This is a manualised intervention which was adapted from a self-help guide called "Life after self-harm" (Schmidt and Davidson 2004) based on the principles of cognitive behavioural therapy. This intervention includes evaluation of the self harm attempt, crisis skills, problem solving and basic cognitive techniques to manage emotions, negative thinking and relapse prevention strategies. This was chosen as there is consistent evidence in literature showing that people who self-harm have poor problem solving skills which may lead to hopelessness and depression.

A multidisciplinary focus group of mental health professionals initially translated the content of the manual into Urdu giving special consideration to cultural adaptation of phrases and concepts to reflect South Asian culture. Culturally appropriate case scenarios were incorporated and a consensual view to address cultural factors such as gender roles, sexuality, and substance misuse and family conflicts were taken. Due to low prevalence of substance misuse in South Asian women, issues related to this were replaced with emphasis on family conflicts.

The intervention will be delivered by qualified mental health professionals who underwent intense group training delivered by a senior psychiatrist. Ongoing training and supervision will be continuing throughout the course of the study.

C-MAPS (Culturally Adapted Manualized Problem Solving Training) will be a brief problem focused therapy comprising of 8 sessions delivered within 3 months after a self-harm episode. We will have two engagement sessions before the actual therapy. The session will involve in-depth explanation of the intervention, will address any concern or anxieties patients may have and will help in building better rapport between the patient and therapist. The adapted therapy will be delivered by therapists in the patient's home/GP practice/hospital depending upon patient's choice. Sessions will be offered weekly in the first month and than fortnightly and will last up to 50 minutes. The treatment will be structured around patient's current problems and helping the patient to deal with specific problems leading to the self harm act. The patients will be able to use this structure and approach in future recurrence.

#### Adaptation of the manual

There is sufficient evidence to suggest that cognitive behaviour therapy might need adaptation for use with ethnic minority clients in the west [21]. It has been suggested that therapists need to be aware of the cultural issues for successfully applying therapy. Assessment needs to take into consideration factors which are relevant to culture and religion of the client. Engagement has been described to be a major barrier in providing therapy in this client group. Finally, therapy techniques might need minor adjustments [22]. Involvement of family can be helpful and therefore family education and involvement where clients are comfortable with this will be tried in all cases. As mentioned above family conflicts are common in this group and therefore an additional session will focus on use of culturally sensitive training in assertiveness and conflict management. The process of adaptation will consist of two steps. In the first step therapists will be trained in culturally sensitive therapy through a workshop. They will also receive supervision throughout the project. The second phase of adaptation will consist of further refinement of manual based on information from our qualitative study.

#### Treatment as usual (TAU) control group

Patients who will be randomized to the "treatment as usual" group will receive routine care. In most cases this consists of an assessment by a casualty doctor or a junior psychiatrist in the emergency department, on the basis of which about one third patients are referred for follow up to psychiatry outpatient, a small number are referred to addiction services, and the remainder are advised to consult their own general practitioner. Patients are not routinely referred to psychotherapy or psychology services [23]. British South Asian self harm patients are more likely to be referred back to their GP as compared to white self harm patients [24]. Participants will receive an initial assessment along with treatment as usual (TAU) as ascertained by the general practitioner or mental health professional. Any type of treatment apart from C-MAPS will be permitted. We will record the details of treatment as usual received and degree of adherence by the patient. Basic demographic information will be recorded for both groups.

### Sample Size

The participants will be randomized to two groups: intervention or treatment-as-usual. A total of thirty participants will be recruited to the pilot. This will ensure that, even after loss to follow-up, we will have at least 10 subjects per group for analysis (FDA guidance http://www.fda.gov/cder/guidance).

### Outcome measures

#### Primary outcome measure

*Beck scale for suicidal ideation: *A 21-item self-report questionnaire used to detect and measure severity of suicide ideation [25]

#### Secondary outcome measures

*Beck hopelessness scale: *Consists of 20 self report questions designed to measure three major aspects of hopelessness; feelings about the future, loss of motivation and expectations [26].

*Beck Depression Inventory: A *21 item self-reported scale to measure the severity of depression [27]

*Euroqol (EQ-5D) Quality *of life - Standardised instrument for use as a measure of health outcome [28] It is a measure of health and quality of life. This is a standardised instrument for use as a measure of health outcome; it provides a simple descriptive profile and a single index value for health status. EQ-5D consists of a self-report questionnaire of five health dimensions (mobility, self-care, usual activities, pain/discomfort and anxiety/depression). We have previously used this instrument with South Asians living in the UK [29]

*Client service receipt inventory (CSRI*) - an estimate of the health and social services received for power calculation (formal and informal such as faith healers). It will also include personal costs e.g. non-attendance at work [30].

*Time to self reported repetition of self harm *(estimate for definitive trial) [31]

*Problem solving Inventory *- Assess individual's perceptions about how they react to and solve personal problems [32]

*Coping resource Inventory *- Assess coping resources available to individual to manage stress [33]

### Translation and Cultural adaptation of outcome measures

Translation: All these scales have also been translated into target languages based on our previous work.

### Qualitative Study

The qualitative component of this study will enable a richer picture to emerge and will contribute to an overall understanding of the key issues relating to self-harm in British South Asian women across the study sites. It will document a range of perspectives with treatment providers and users [34]

### Aims and purpose

The aim of the qualitative study is to explore the experience of the patients and therapists to further refine the therapy manual.

We will carry out qualitative in depth interviews with those who complete the intervention and those who drop out of therapy. This is a central part of the qualitative study as we consider the experiences of British South Asian women in contact with services as crucial to more effective and sensitive services [35]

Additionally focus groups will be conducted with the therapists to triangulate data. The aim of these interviews and focus group (s) will be

1. To see what works in therapy and what does not work

2. Which part of therapy the patients find useful

3. Which parts/techniques they don't find too helpful or too alien and which should be addressed in future work for further adaptation

4. Their overall experience of the service and treatment

### Study Participants

1. **Group 1: **Patients who have completed therapy will be interviewed to explore their experience of therapy. This will help us to understand patient's perception of therapy, the techniques they found useful and techniques that were not very helpful. We will also ask them whether there was anything outside therapy they found useful to see if it can be incorporated into therapy. These interviews will further explore the characteristics in therapist they found were helpful or unhelpful. Interviews will be conducted until data saturates, however, we will stop interviews after 7-8 patients have been interviewed due to resource issues. Based on our previous experience however we feel that this number will give us sufficient information to further refine our manual. These interviews will also explore the pathways to care and treatment experience of this group in order to see whether that can be taken into consideration to further improve engagement in therapy and treatment in general.

2. **Group 2: **Patients who drop out of therapy will be asked for their permission to be interviewed. Those who agree will be interviewed to explore the reasons for dropping out of therapy and whether they found anything useful and what could be done to improve engagement with therapy. We expect a number similar to the first group will be required in this group.

3. **Group 3: **This group will consist of mental health professionals who have provided therapy to patients. The idea is to explore their experience of providing therapy, what did they think work and what need to be changed for further improving assessment, engagement and making a positive change in patients lives in future. We will conduct a focus group with these therapists. Therapists will be encouraged to write regular diaries as field notes of any difficulties and this information will be used to further enhance our understanding. These focus groups will further explore their experiences of delivering the intervention; perceptions of gaps in the manual/delivery, what skills they think are needed for effective delivery, perceptions of the impact of race/gender/age of therapist on the intervention. This set of interviews will also help to identify the kinds of difficulties encountered by health care staff in working with this group of patients, and make recommendations to improve services.

### Procedure

Interviews will be semi structured and will be held in English where possible and in a culturally sensitive manner. In cases where interviews are conducted in Urdu, Hindi, Punjabi transcripts will be translated in English language. Interviews are considered as more appropriate for patients due to the high stigma attached to mental illness in this group, and based on our previous work, which suggests that participants of group might hesitate in sharing their experiences. Therapists will be asked to share their opinion through focus group. This is because this is a homogenous group and will be more cost effective. Additional information gathered through the field's notes and supervision notes will also be included in final analyses to guide our final refinements of the manual. Interviews and focus groups will be conducted using interview guidelines; however, we will keep an open mind and explore any newer ideas, which arise during the interviews or focus groups if they are considered to be relevant. This will help us in keeping our focus on the main aim of this part of the study, i.e., refinement of the therapy manual.

These three qualitative elements of the study will enable a much broader picture of the issues to emerge and will complement the quantitative component.

#### Data Analysis

Each interviewer will start analyzing the interviews as and when they are conducted. Analysis involves the researcher immersing him in data by reading the interview transcripts carefully several times, and identifying emerging themes and categories [36]. Where required, interviewers will contact the participants by phone or in person to clarify points and emergent themes that arise from the initial transcriptions or analysis. Interviewers will meet regularly for supervision and support throughout the study. During these meetings emerging themes, concepts and conflicts will be discussed. Interview transcripts will help us in guiding further interviews to clarify themes. This process will be repeated, thus modifying the working codebook till a final set of codes will be obtained. The process will stop when saturation point is reached and we realize that no new themes are emerging.

##### Qualitative analysis

A thematic content analysis method will be used to analyze the transcribed interviews following a systematic, iterative process. Codes will be applied to the transcripts, and converted into categories to represent the main themes arising from the data [37]. A small number of interviews will be analyzed independently by two other members of the research team. Themes emerging from the analyses of interviews by independent researchers will be compared and contrasted with others constantly. Triangulation of themes and concepts will be used to further compare and contrast the data from the different participating groups. This will enhance the reliability and validity of the analysis.

##### Quantitative Analysis

An intention to treat analysis will be carried out. Both parametric and non-parametric tests will be used for data analysis. Mean and standard deviation scores for all continuous measures shall be presented, and normality will be assessed using Kolmogrov-smirnov test for normality. Spearman's correlation coefficient shall be used to measure associations among different measures. Baseline differences will be measured using a t test for normative data and Chi square test for categorical data. This will enable us to take into account any baseline differences in our final analyses. Differences between the treatment and the control group will be measured using linear regression, while controlling for baseline differences.

## Discussion

Research shows increased risk of suicide and attempted suicide among British South Asian women in the UK. There are a number of psychosocial treatments available for self harm but to the best of our knowledge there has been no trial of any evidence based intervention in this hard to reach group. As this is a complex intervention so we are proposing a phased approach to the development. We aim to carry out this feasibility trial to find out if this culturally adapted psychological intervention compared with treatment as usual results in decreasing hopelessness and suicidal ideation.

The impact of this intervention on service users will be to improve their mental health literacy and stress management capabilities through problem solving and cognitive restructuring. This may lead to reduction of further self harm, better health status and improved satisfaction with health services.

We are undertaking a detailed process of culturally tailoring this intervention to the specific needs of our target population. This will not only help in improved efficacy but will possibly lead to fewer dropouts and higher satisfaction.

The outcome of this trial and subsequent definitive trial will benefit NHS, in terms of helping to reduce suicide rates and provide better care for self harming British south Asian women. We expect the results to help guide development of better services and tailoring existing services to needs of hard to reach vulnerable groups.

## List of Abbreviations

C-MAP: culturally adapted manualized problem solving; CSRI: Client Service Receipt Inventory; EQ-5D: EuroQol-5Dimensions; FDA: Food and Drug Administration; GP: General Practitioner; RCT: Randomised Controlled Trial; REC: Research Ethics Committee; SRQ: Self rating Questionnaire; TAU: Treatment as Usual; UK: United Kingdom.

## Competing interests

The authors declare that they have no competing interests.

## Authors' contributions

NH, WW & IC conceived the idea for the study, NC, SVD, SK, DN, MH all contributed in the translation of the Manual and running of the focus groups. SVD & SK have done the baseline assessments so far. All authors except SK will also be providing the intervention. FN contributed in the development of Qualitative methodology. All authors read and approved the final manuscript.
